# Return visit characteristics of SARS-CoV-2 PCR-positive cases in a pediatric emergency department

**DOI:** 10.3906/sag-2102-281

**Published:** 2022-01-25

**Authors:** Nihan ŞIK, Durgül ÖZDEMİR, Murat DUMAN

**Affiliations:** Division of Pediatric Emergency Care, Department of Pediatrics, Dokuz Eylül University Faculty of Medicine, İzmir, Turkey

**Keywords:** Children, COVID-19, SARS-CoV-2, return, emergency department, pandemic

## Abstract

**Background/aim:**

The aim of this study was to evaluate return visits to the pediatric emergency department (ED) for children who were detected to be positive for SARS-CoV-2 by polymerase chain reaction (PCR).

**Materials and methods:**

Between April 2, 2020, and January 20, 2021, children aged 0 to 18 years who were detected to be SARS-CoV-2 PCR-positive and discharged from the ED were evaluated. Among them, patients who returned to the ED within 14 days of quarantine were included in the study. For the first presentation and return visit, demographics, clinical findings, laboratory and radiologic investigations, and ward/pediatric intensive care unit (PICU) admissions were recorded. Patients were divided into 5 groups according to clinical severity.

**Results:**

Among 575 children who were confirmed to be SARS-CoV-2 PCR-positive, 50 (8.6%) of them [median age: 10.4 years (IQR: 4.8–15.2); 26 females] had returned. There was no difference for age, sex, underlying diseases, or symptoms for patients who returned or did not for the first presentation, but the percentage of those from whom laboratory tests were obtained was higher in cases of return visits. For symptomatic cases on the first presentation, the most common reason for return was having additional symptoms. The most common symptoms at the return visit were fever, cough, and sore throat. There was no severe/critical case in terms of clinical severity. Among all cases, 36 (72.0%) patients were discharged from the ED, 13 (26.0%) were observed for 6–8 h and then discharged, and 1 (2.0%) was admitted to the ward; there was no PICU admission or death, respectively.

**Conclusion:**

Patients who returned to the ED had mild clinical presentations. Understanding the frequency of and risk factors for return visits can clarify public health priorities such as healthcare planning to ensure the availability of resources needed for acute and follow-up care of children with COVID-19.

## 1. Introduction

Coronavirus disease 2019 (COVID-19), caused by severe acute respiratory syndrome coronavirus-2 (SARS-CoV-2), has rapidly evolved into a pandemic and has been an emerging disease of global public health concern. As it is regarded as a pandemic, huge challenges still exist for global prevention and control strategies [1,2]. COVID-19 has caused an unprecedented health crisis worldwide. There has been substantial pressure on healthcare systems to meet the escalating demands from the swelling pandemic surge [3].

As the incubation period ranges between 1 and 14 days, many of the COVID-19 patients who are discharged are recommended to undergo a 14-day quarantine and treatment at home [4–6]. However, some patients with COVID-19 may develop serious illness several days after the initial symptoms [7]. Limited data exist on whether these patients fully recover or if they re-present to the emergency department (ED) [4–6]. Return visits of patients with COVID-19 disease have been a common and costly public health concern that endangers patient safety and may further drain hospital resources during this public health emergency period. Understanding the associations of COVID-19 with return visits may have useful implications for policy-making in an effort to optimize healthcare delivery [8]. Concerns for surges in hospital occupancy force emergency providers to preserve inpatient resources [7]. Published data mostly include information on the clinical course, laboratory and radiologic results, and treatment of patients with COVID-19 [4–6]. However, to date, there is a relative paucity of data on studies focusing on representations of COVID-19 patients.

The aim of this study was to evaluate characteristics of return visits to the pediatric ED within 14 days of discharge for children who were detected to be SARS-CoV-2 polymerase chain reaction (PCR)-positive.

## 2. Materials and methods

### 2.1. Study design

This was a single-center retrospective study performed in the pediatric ED of a tertiary hospital with approximately 120,000 pediatric emergency department visits per annum. The study was approved by the local ethics committee (approval number: 2020/30-03).

The study population included children aged 0 to 18 years who presented to the pediatric ED and were diagnosed as SARS-CoV-2 reverse-transcription PCR-positive with a confirmed nasopharyngeal specimen based on the guidelines published by the Ministry of Health’s Scientific Committee, then discharged from the ED with quarantine recommendations. Among them, those who returned to the pediatric ED within 14 days of quarantine were included in the study.

We used International Classification of Diseases codes for COVID-19 to identify patients. We obtained information from a computer database and electronic medical records. Patients who were diagnosed with COVID-19 in another facility, those for whom a control visit was planned by our medical staff after discharge from the ED, and those with insufficient data were excluded.

For the first presentation and the return visit, demographics, presence of chronic illness, symptoms with duration, history of contact with suspected/confirmed COVID-19 cases, and the presence of an individual in the patient’s household in quarantine, hospitalized in a ward/intensive care unit, or who had died were recorded. The time between the first presentation and return was calculated. Because symptoms of sore throat or smell/taste loss cannot be described by infants and preschool-aged children, patients over the age of 3 years were asked about sore throat, while patients of 5 years and older were asked about smell/taste loss. Cases were divided into 4 age groups as ≤1 year, 1–6 years, 6–10 years, and >10 years. Clinical findings, laboratory data, radiologic investigations, and diagnoses for both the first presentation and the return visit were recorded. According to clinical severity, patients were divided into 5 groups as asymptomatic, mild, moderate, severe, or critical as previously described [9]. In addition, patients did not receive specific treatment for COVID-19.

Reasons for return were divided into 5 groups as follows: Group 1: Those who had additional symptoms while being symptomatic at the time of the first presentation. Group 2: Those who became symptomatic while being asymptomatic at the first presentation. Group 3: Those who had aggravation of symptoms without any different symptoms from the time when the first presentation occurred. Group 4: Those with ongoing symptoms, without aggravation or any additional symptoms. Group 5: Any other reason.

For the return visit, patients were divided into 3 groups according to the final decision that was made: Group 1: Discharged from the ED. Group 2: Observed for 6–8 h in the ED and then discharged. Group 3: Admitted to the hospital [ward/pediatric intensive care unit (PICU)]. Finally, the need for respiratory support, length of stay in the hospital, and prognosis were recorded.

### 2.2. Statistical analysis

All statistical analyses were performed using SPSS 22.0 software for Windows (IBM Corp., Armonk, NY, USA). Categorical and continuous variables were reported as frequencies and percentiles and as means with standard deviations (SDs) or medians with interquartile ranges (IQRs). The Mann-Whitney U test was used to compare nonparametric data and student’s t-test was used for parametric data. A value of p < 0.05 was considered statistically significant.

## 3. Results

During the study period, 575 children were confirmed to be PCR-positive for SARS-CoV-2 and were discharged from the pediatric ED. Among them, 50 (8.6%) returned to the ED within 14 days of quarantine. There was no difference for age, sex, history of contact with suspected/confirmed COVID-19 cases, underlying diseases, symptoms, or radiologic investigations for patients who returned or did not follow the first presentation, but the percentage of those from whom laboratory tests were obtained was higher for patients who returned to the ED, as shown in Table 1.

Twenty-six (52.0%) of the patients were female and the median age was 10.4 years (IQR: 4.8–15.2). The most common age group was >10 years with 28 (56.0%) patients, followed by 1–6 years (n: 12, 24.0%) and 6–10 years (n: 10, 20.0%); there was no patient under the age of 1 year. Nine (18.0%) of the patients had chronic illnesses, as shown in Table 2. The time between the first presentation and return visit was a median of 6.0 days (IQR: 2.7–10.0), as shown in Table 3.

Evaluating the first presentation of these patients, 36 (72.0%) were symptomatic and the most common symptom was fever (n: 19, 38.0%), followed by cough (n: 9, 18.0%), sore throat (n: 9, 18.0%), and fatigue (n: 9, 18.0%). Twenty-nine (58.0%) of them had contact with a SARS-CoV-2 PCR-positive individual who was symptomatic in 28 (96.5%) cases and a household member in 26 (89.6%) cases. Among household individuals, 47 (94.0%) were in quarantine and 3 (6.0%) had been admitted to the ward. The diagnosis was upper respiratory tract infection in 31 (62.0%) cases and acute gastroenteritis in 5 (10.0%). Laboratory tests were obtained from 14 (28.0%) patients and radiologic investigations were performed for 11 (22.0%), the latter being chest X-ray for 10 (20.0%) patients and computed tomography (CT) for 1 (2.0%) patient, as shown in Table 2.

Evaluating return visits, the most common reason was additional symptoms for cases that were symptomatic at the time of the first presentation with 29 (58.0%) patients. There were 9 (18.0%) patients who became symptomatic while being asymptomatic at the first presentation and 8 (16.0%) patients who had aggravation of symptoms without any different symptoms from the time when the first presentation occurred. Three (6.0%) patients returned with ongoing symptoms, without aggravation or any additional symptom. Finally, there was 1 patient with an “other” reason: a 6-year-old boy with hemophilia who had fallen and needed factor replacement. The family was usually able to provide factor treatment intravenously at home, but he was admitted to the ED because of absence of intravenous access.

The most common symptoms at return visits were fever (n: 15, 30.0%), cough (n: 9, 18.0%), and sore throat (n: 8, 16.0%). The median time for onset of new symptoms or aggravation of symptoms was 2.0 days (IQR: 1.0–2.0). Laboratory tests were obtained in 30 (60.0%) cases and radiologic investigations were performed in 19 (38.0%) cases, the latter being chest X-ray for 17 (34.0%) patients and CT for 2 (4.0%) patients. Compared with the first presentation, there was an increase in the percentage of patients from whom laboratory tests or radiologic investigations were obtained for return visits. For 26 (52.0%) patients, the diagnosis was the same as in the first presentation. Additionally, 13 (26.0%) patients were diagnosed with upper respiratory tract infection, 5 (10.0%) with anxiety, and 4 (8.0%) with acute gastroenteritis. All patients with anxiety were adolescents aged between 15 and 17 years old. For 6 (12.0%) patients, more than 1 return visit occurred. According to clinical severity, 48 (96.0%) of the patients were in the mild and 2 (4.0%) in the moderate group; there was no severe or critical case in terms of clinical severity.

There was no need for respiratory support for any patient in return visits. Of all patients, 36 (72.0%) were discharged from the pediatric ED, 13 (26.0%) were observed for 6–8 h and then discharged, and 1 (2.0%) was admitted to the ward; there was no PICU admission, as shown in Table 3. The patient admitted to the ward was a 3-year-old boy with Wilms tumor who had a runny nose upon the first presentation to the ED. On the 10^th^ day of the quarantine, he had developed neutropenic fever, so he was admitted to the ward and piperacillin-tazobactam was started. He was discharged without any complications. No patients died during the return visit period.

## 4. Discussion

The COVID-19 pandemic has led to catastrophic effects for global health worldwide. Many healthcare facilities remain stretched beyond their capacity. Return visits to EDs have the potential to exacerbate this burden and may represent missed opportunities to provide optimal care [10]. Understanding the epidemiology of returns among COVID-19 patients would allow the healthcare system to focus its already limited resources and may improve outcomes during such a pandemic [11].

To the best of our knowledge, this is the first study evaluating return visit characteristics of children with COVID-19. Although limited, adult studies exist in the literature that have evaluated returns to EDs or ward/intensive care unit readmissions of COVID-19 patients. Discussing return visits for children with COVID-19 is challenging, as we do not yet have enough information on the clinical characteristics of the disease or established treatment regimens and care bundles. Therefore, it is difficult to analyze and discuss the factors contributing to the occurrence of return visits for the pediatric population.

Ye et al. reported that 11.0% of adults had returned to the ED within 14 days and, of these, 7.6% were readmitted to the hospital [12]. In a Spanish study, it was reported that 20.5% of discharged patients revisited the ED, mainly for persistence or progression of symptoms, and among them, 38.8% were hospitalized [13]. We found a return visit rate of 8.6% for children at a median of 6 days after discharge from the ED and, for 12.0% of the patients, returns occurred more than once.

Among published studies, the most common reasons for returns were respiratory distress, pain, altered mental status, falls, fever, soft tissue infection, thrombotic events, and gastrointestinal symptoms [6,10,14–17]. In a Spanish study, ED revisits were associated with a history of rheumatologic disease, digestive symptoms, a respiratory rate of >20 breaths/min, and corticosteroid therapy given in the emergency department; in addition, age of >48 years and fever were associated with hospitalization after ED readmission [13]. Kilaru et al. reported that age, abnormal chest X-ray findings, and fever or hypoxia on presentation were independently associated with an increased rate of return to the ED [7]. A recent analysis found chronic conditions to be associated with hospital readmissions, which could be explained by the complications of underlying diseases in the presence of COVID-19 [15]. The most common reasons for readmission were reported as respiratory distress and thrombotic episodes in another analysis, while those happening at a later time (>12 days after discharge) included exacerbations of psychiatric illness and falls [3]. Somani et al. reported that rates of intensive care unit admission and death were 5.8% and 3.6% on returns. In our study, the most common reason was having additional symptoms for cases symptomatic at the time of the first presentation and the most common symptoms were fever, cough, fatigue, and sore throat. There was no need for respiratory support and no severe/critical case in our study, and only one patient was admitted to the ward; there was also no PICU admission or death. This could be related to the fact that children usually have a milder clinical course than adults, which seems to continue holding true for return visits. In our study, the percentage of those from whom laboratory tests were obtained was higher among patients who returned to the ED, but there was no difference for initial symptoms, age, or percentage of chronic diseases between those who returned and those who did not. Cases for which laboratory tests were obtained were not severe/critical at the first presentation or for the return visit. In addition, compared with the first presentation, there was an increase in the percentage of patients for whom laboratory tests or radiologic investigations were performed for return visits. Hence, further examinations at the first presentation may have increased the anxiety levels of these patients; more detailed information and support should be provided to patients about the postdischarge process. Increased rates of laboratory or radiologic tests may also pose a risk of increased workload for EDs and healthcare costs. This is important for healthcare planning to ensure the availability of resources needed during a pandemic.

In adult studies, it was suggested that at least 20% of patients had elevated anxiety and depressive symptoms after hospitalization for pneumonia and acute coronary syndromes [18,19]. Ye et al. found that 10% of patients screened positive for anxiety, depression, and loneliness after discharge [12]. Likewise, 10.0% of our patients returned to the ED with anxiety; all of these were adolescents who did not have any known psychiatric disorders and had not experienced anxiety before. A higher level of care can be provided at home for these patients. During the COVID-19 pandemic, it was reported that patients were more likely to call their primary care providers or the hospital helpline before deciding to seek care in EDs [9]. Therefore, implementation of telemedicine in ED practice to decrease the anxiety might be an important point for those with elevated anxiety.

Patients may have an unplanned return for any reason, and the uncertain natural history of the disease may make it difficult for emergency providers to predict which patients will worsen among those who initially appear well [7]. Hence, knowledge about the characteristics of patients who are most at risk of return could help to better decide when to discharge patients and how to select those who need closer follow-up after discharge [16]. Meanwhile, return hospital visits do not equate to a failure inpatient care; rather, this outcome represents the need for a higher level of care than can be provided at home [7]. Risk stratification may further improve the efficacy of home monitoring and telemedicine services by focusing attention on patients at higher risk of deterioration [7].

It should be noted that the learning curve for COVID-19 has changed over the course of the pandemic, which may have had a varying impact on patient discharge. Concerns about gaps in postdischarge care for COVID-19 patients and an uncertain clinical course have had the potential to delay hospital discharges [12]. There were previously limited published guidelines for safe discharge parameters for COVID-19 patients and few known risk factors for return visits [12]. We need to be alert to the fact that these patients could infect other people during return processes. Reducing preventable ED revisits may draw policy attention as an opportunity to improve the quality of care and reduce healthcare costs [20].

We acknowledge the limitations of our study. The number of patients who returned was limited. Furthermore, during the study period, there was no strict protocol for hospitalization or discharge of the patients; the decision was reserved for the judgment of individual physicians.

In conclusion, patients who returned to the ED had a mild clinical presentation and there was no need for respiratory support and no PICU admission or mortality; there was also an increase in the percentage of patients for whom laboratory or radiologic tests were performed during return visits. Understanding the frequency of and risk factors for return visits can help shape public health priorities such as healthcare planning to ensure the availability of resources needed for acute and follow-up care of children with COVID-19.

## Figures and Tables

**Table 1:** Demographics, clinical findings, and presence of laboratory/radiological investigations of the patients who returned or not in the study.

Parameter	Return visit (+) n: 50	Return visit (−) n: 525	p
**Age in years, median (IQR)**	10.4 (4.8–15.2)	12.0 (4.2–15.1)	0.616
**Female sex, n (%)**	26 (52.0)	280 (53.3)	0.477
**Contact with a COVID-19 PCR-Positive individual, n (%)**	29 (58.0)	307 (58.4)	0.560
**Underlying disease, n (%)**	9 (18.0)	55 (10.5)	0.185
**Presence of any symptom at first admission, n (%)**	36 (72.0%)	414 (78.8)	0.401
**Laboratory investigations at first admission, n (%)**	25 (50.0)	167 (31.8)	0.005
**Lymphocyte count/mm3, median (IQR)**	2300 (1800–2900)	1300 82100–2900)	0.576
**C-reactive protein (mg/L), median (IQR)**	1.3 (0.7–4.0)	4.1 (1.0–10.5)	0.043
**Procalcitonin (ng/mL), median (IQR)**	0.03 (0.01–0.04)	0.05 (0.02–0.10)	0.016
**Radiological investigations at first admission, n (%)**	11 (22.0)	123 (23.4)	0.137

IQR: Interquartile range

**Table 2:** Demographics, history of contact with a COVID-19-positive individual, and clinical findings of the patients for the first presentation to the pediatric emergency department.

Variable	n: 50
**Female sex, n (%)**	26 (52.0)
**Age in years, median (IQR)**	10.4 (IQR: 4.8–15.2)
**Age group, n (%)**	
0–1 year	-
1–6 years	12 (24.0)
6–10 years	10 (20.0)
>10 years	28 (56.0)
**Underlying disease, n (%)**	9 (18.0)
**Contact with a COVID-19 PCR-positive individual, n (%)**	29 (58.0)
In-house, n (%)	26 (76.6)
**Symptoms, n (%)**	
Fever	19 (38.0)
Cough	9 (18.0)
Fatigue	9 (18.0)
Sore throat	9 (18.0)
Runny nose	6 (12.0)
Abdominal pain	4 (8.0)
Taste/smell loss	4 (8.0)
Headache	4 (8.0)
Diarrhea	2 (4.0)
Nausea/vomiting	2 (4.0)
**Households, n (%)**	
In quarantine	47 (92.0)
Admitted to the ward	3 (6.0)
Admitted to the intensive care unit	-
Exitus	-
**Laboratory tests, n (%)**	14 (28.0)
**Radiologic investigations, n (%)**	11 (22.0)
Chest X-ray	10 (20.0)
Chest computed tomography	1 (2.0)
**Diagnosis, n (%)**	
Asymptomatic infection	14 (28.0)
Upper respiratory tract infection	31 (62.0)
Acute gastroenteritis	5 (10.0)

IQR: Interquartile range.

**Table 3:** Return visit characteristics of the patients in the study.

Variable	n: 50
**The time between first presentation and return visit (days), median (IQR)**	6.0 (2.7–10.0)
**More than one return visit, n (%)**	6 (12.0)
**Reason for a return visit, n (%)**	
Had additional symptoms while being symptomatic at the first presentation	29 (58.0)
Became symptomatic while being asymptomatic at the first presentation	9 (18.0)
Aggravation of symptoms without any different symptoms from the time when the first presentation occurred	8 (16.0)
Ongoing symptoms without aggravation or any additional symptoms	3 (6.0)
Other	1 (2.0)
**Symptoms, n (%)**	
Fever	15 (30.0)
Cough	9 (18.0)
Sore throat	8 (16.0)
Fatigue	6 (12.0)
Abdominal pain	6 (12.0)
Shortness of breath	6 (12.0)
Diarrhea	5 (10.0)
Chest pain	4 (8.0)
Headache	3 (6.0)
Nausea/vomiting	3 (6.0)
Palpitation	3 (6.0)
Runny nose	3 (6.0)
Taste/smell loss	2 (4.0)
Rash	2 (4.0)
**Time for the onset of new symptoms or aggravation of symptoms (days), median (IQR)**	2.0 (1.0–2.0)
**Laboratory tests, n (%)**	30 (60.0)
**Radiologic investigations, n (%)**	19 (38.0)
Chest X-ray	17 (34.0)
Chest computed tomography	2 (4.0)
**Diagnosis, n (%)**	
Same as the first presentation	26 (52.0)
Upper respiratory tract infection	13 (26.0)
Psychiatric aggravation	5 (10.0)
Acute gastroenteritis	4 (8.0)
**Clinical severity, n (%)**	
Mild	48 (96.0)
Moderate	2 (4.0)
Severe/critical	-
**The final decision, n (%)**	
Discharged from the pediatric emergency department	36 (72.0)
Observed for 6–8 h and then discharged	13 (26.0)
Admitted to the ward	1 (2.0)
Admitted to the PICU	-

IQR: Interquartile range, PICU: Pediatric intensive care unit.
